# Submucosal tunneling endoscopic resection technique with intermuscular dissection for a rectal gastrointestinal stromal tumor

**DOI:** 10.1055/a-2318-3050

**Published:** 2024-05-29

**Authors:** Salvatore Russo, Silvia Cocca, Flavia Pigò, Giuseppe Grande, Stefania Caramaschi, Rita Conigliaro, Helga Bertani

**Affiliations:** 1 Gastroenterology and Digestive Endoscopy Unit, Modena University Hospital, Modena, Italy; 2 9306 Department of Medical and Surgical Sciences for Children and Adults, Anatomic Pathology Unit, University of Modena and Reggio Emilia, Modena, Italy

A 54-year-old woman was referred to our center for evaluation of a subepithelial tumor located in the posterior wall of the rectum, 1 cm proximal to the anal verge. Endoscopic ultrasonography showed a 15-mm hypoechoic homogeneous submucosal lesion. Submucosal tunneling endoscopic resection (STER) was performed ( Video 1 ) under monitored anesthesia care, using CO _2_ insufflation, a standard gastroscope (GIF-H190) with a transparent cap (D-201-10704), DualKnife J 1.5 mm (Olympus, Tokyo, Japan), and a VIO 200D (Erbe, Tübingen, Germany). The submucosal lift was achieved with a mixture of saline solution and indigo carmine. A small horizontal incision was made at the distal margin and a submucosal pocket was created. After dissecting the subepithelial tumor from the submucosa ( Fig. 1 ), an intermuscular dissection assisted by a water-jet injection into the intermuscular space was performed ( Fig. 2 , Fig. 3 ). Finally, the larger vessels were coagulated with a bipolar forceps (HS-D2622; Pentax, Tokyo, Japan) and the mucosal defect was closed with four 11-mm through-the-scope clips (MED-204-CLP; Meditalia, Palermo, Italy). The technical duration of the procedure was 30 minutes. The patient was discharged 2 hours later with the indication to take prophylactic oral antibiotic therapy for 5 days. No complications were reported. Histology showed a 12-mm gastrointestinal stromal tumor (GIST) surrounded by thin smooth tissue, with free margins (R0) and mitotic index <5/mm ^2^ ( Fig. 4 ).

**Fig. 1:**
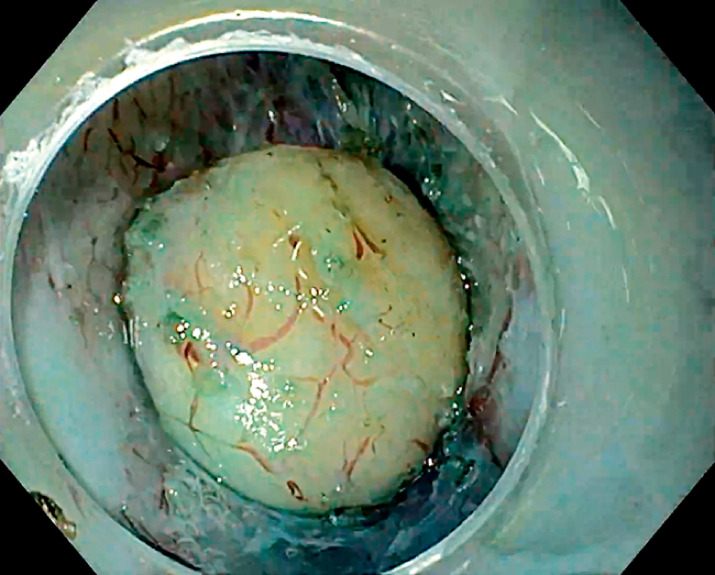
GI stromal tumor in the tunnel after submucosal dissection was completed.

**Fig. 2:**
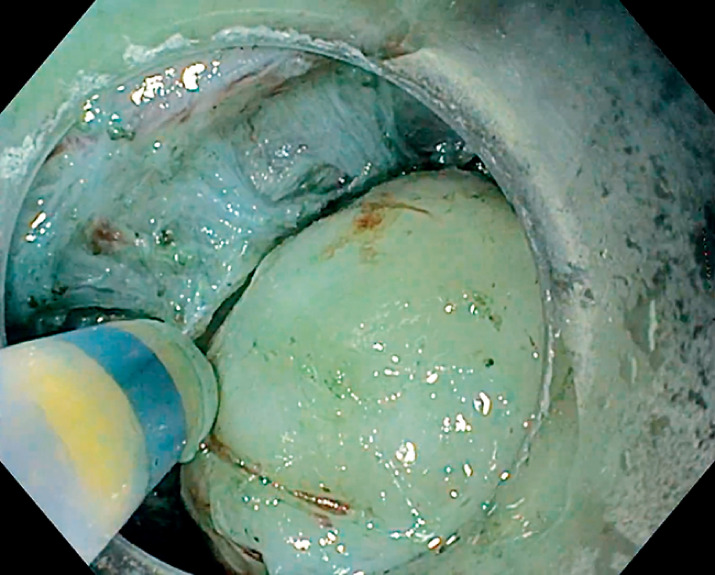
Intermuscular dissection.

**Fig. 3:**
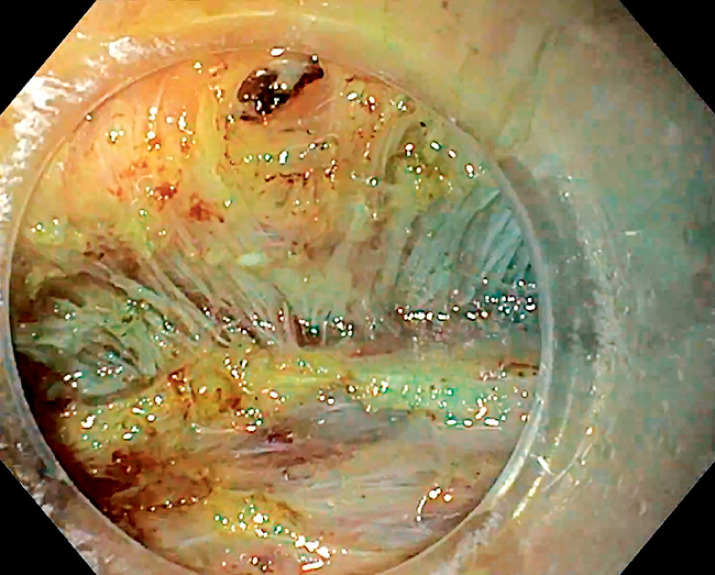
Resection base showing integrity of the external longitudinal muscle.

**Fig. 4:**
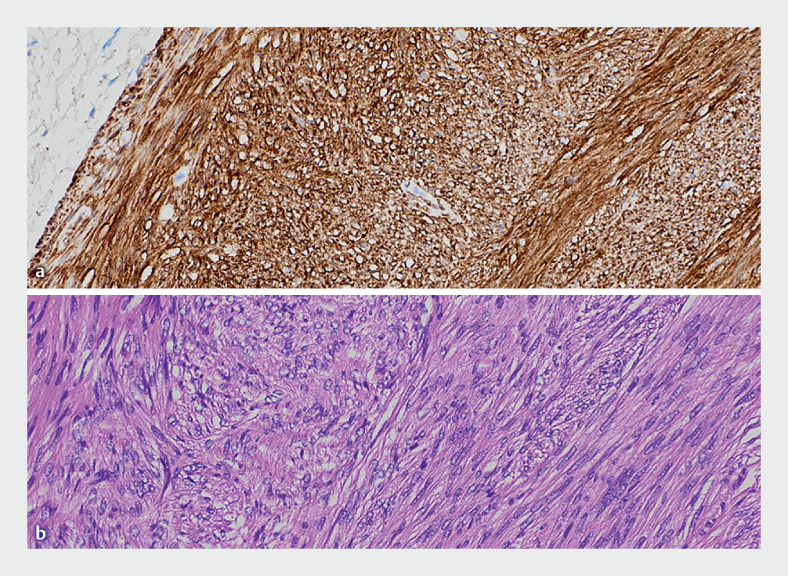
Histological features. **a** DOG1 marker (immunohistochemistry, 20×). **b** Hematoxylin and eosin (20×); spindle cells, absence of necrosis.

Submucosal tunneling endoscopic resection technique with intermuscular dissection for a rectal gastrointestinal stromal tumor.Video 1

After multidisciplinary consultation, a chest and abdominal computed tomography (CT) scan with intravenous contrast was performed, which showed no pathological findings, and a postoperative follow-up observation was scheduled considering the extremely low risk of recurrence.

GISTs are rare and account for 0.6% of all rectal neoplasias 1 . To date, the best treatment regimen remains uncertain 2 and data on endoscopic resection of these tumors are scarce 3 4 5 . STER is emerging as a less invasive alternative to surgery for subepithelial tumors in the upper gastrointestinal tract 2 and it also seems safe and effective to treat carefully selected rectal GISTs.

Endoscopy_UCTN_Code_TTT_1AQ_2AD_3AZ

## Notes

Endoscopy E-Videos https://eref.thieme.de/e-videos
*E-Videos* is an open access online section of the journal *Endoscopy* , reporting on interesting cases and new techniques in gastroenterological endoscopy. All papers include a high-quality video and are published with a Creative Commons CC-BY license. Endoscopy E-Videos qualify for HINARI discounts and waivers and eligibility is automatically checked during the submission process. We grant 100% waivers to articles whose corresponding authors are based in Group A countries and 50% waivers to those who are based in Group B countries as classified by Research4Life (see: https://www.research4life.org/access/eligibility/ ). This section has its own submission website at https://mc.manuscriptcentral.com/e-videos .
